# Effects of probiotics on gastrointestinal complications and nutritional status of postoperative patients with esophageal cancer: A protocol of randomized controlled trial

**DOI:** 10.1097/MD.0000000000025138

**Published:** 2021-03-19

**Authors:** Chao Liu, Jing Yang, Weiwei Dong, Jinyan Yuan

**Affiliations:** a The NO.2 Hospital of Baoding Department of Gastroenterology; b Shanxi Provincial People's Hospital Thoracic Surgery.

**Keywords:** complications, esophageal cancer, nutritional status, probiotics, protocol, Randomized controlled trial

## Abstract

**Background:**

Gastrointestinal complications and malnutrition are common problems that affect postoperative rehabilitation and survival of patients with esophageal cancer. Evidence has shown that probiotics have a positive effect on improving gastrointestinal complications and nutritional status of patients with esophageal cancer after surgery, but there is a lack of prospective studies on this topic. We designed this prospective randomized controlled trial to evaluate the effects of probiotics on gastrointestinal complications and nutritional status in patients with postoperative esophageal cancer.

**Methods:**

This is a prospective, randomized, double-blind, placebo-controlled trial. It was approved by the Clinical Research Ethics Committee of our hospital. 192 patients will be randomly divided into probiotics group and the placebo group in a 1:1 ratio. After operation, probiotics and placebo will be given orally for 8 weeks. The indexes of nutritional status and incidence of digestive tract complications will be recorded and the data will be analyzed by SPSS 18.0 software.

**Discussion:**

This study will evaluate the effect of probiotics on gastrointestinal complications and nutritional status of postoperative patients with esophageal cancer. The results of this study will provide clinical basis for the use of probiotics in postoperative treatment of esophageal cancer.

**Trial registration:**

OSF Registration number: D DOI 10.17605/OSF.IO/QHW86

## 1 Introduction

As many as 80% of esophageal cancer patients are malnourished.^[1]^ Due to the particularity of tumor location, insufficient nutrition intake and malignant consumption of tumor, negative effects of patients’ bad psychological state (such as depression, anxiety, fear, etc.) can lead to negative nutritional balance in patients with esophageal cancer.^[2,3]^ At present, the treatment of esophageal cancer is still based on surgery combined with radiotherapy and chemotherapy. Postoperative changes in the anatomical pathway of the upper digestive tract, perioperative fasting (usually 5–7 days), surgical trauma stress, absorption of endotoxin and destruction of mucosal barrier lead to the disorder of digestive tract flora, resulting in a series of gastrointestinal complications and aggravating the poor nutritional status of postoperative patients with esophageal cancer.^[4]^ It is not conducive to postoperative rehabilitation of patients,^[5]^ and limits further anti-tumor therapy such as radiotherapy and chemotherapy at the same time, which has a serious impact on the prognosis and survival of patients.^[6,7]^ Therefore, gastrointestinal complications are the most important perioperative complications of esophageal cancer. It is particularly important to reduce the incidence and improve the nutritional status after operation.

There are many kinds of colonizing bacteria in the human digestive tract, and these colonizing bacteria cluster together to form a colony.^[8]^ Existing studies have confirmed that changes in the composition of digestive tract flora are closely related to esophageal cancer and digestive tract carcinogenesis.^[8–10]^ Normal alimentary tract flora plays an important role in host nutrient metabolism, drug metabolism, maintenance of intestinal mucosal barrier structural integrity, immune regulation, etc. It has been shown that the supplementation of probiotics can adjust the composition of the maladjusted flora in the body, maintain the microecological balance, and promote the recovery and health of the body.^[11]^ At present, probiotics have been widely used in the postoperative adjuvant therapy of digestive system tumors such as colorectal cancer and gastric cancer, and have been proven to reduce complications, improve the integrity of the intestinal mucosal barrier, and improve the nutritional status.^[12]^ Although retrospective studies have confirmed that probiotics are beneficial for patients with esophageal cancer,^[13]^ no rigorous clinical studies have evaluated the effects of probiotics on nutrition and gastrointestinal complications in patients with postoperative esophageal cancer.

Therefore, we will conduct a prospective randomized controlled study to analyze whether postoperative intake of probiotics in patients with esophageal cancer can reduce gastrointestinal complications and improve the nutritional status of patients.

## 2 Materials and methods

### 2.1 Study design

**Figure 1:**
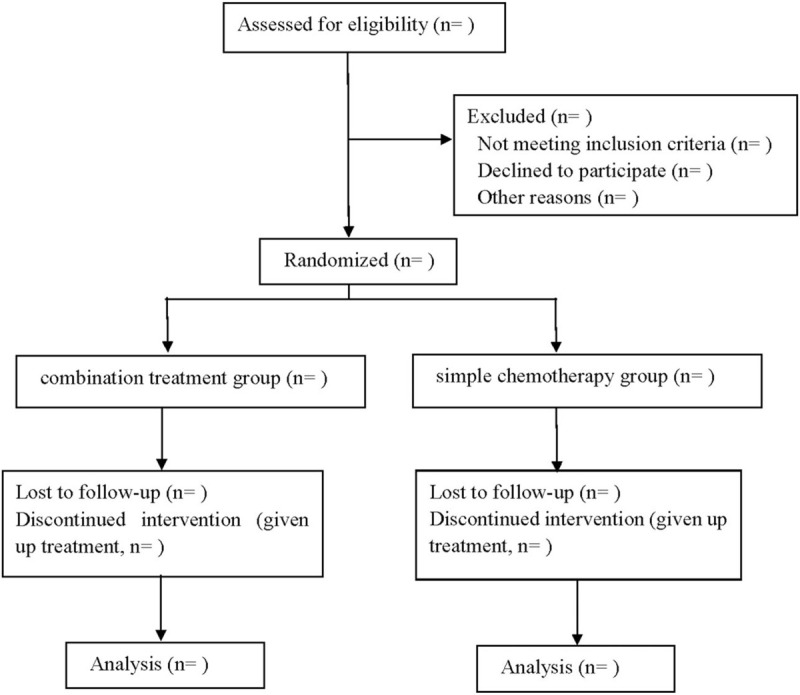
Flow diagram.

This is a randomized, double-blind, placebo-controlled prospective trial to study the effects of probiotics on gastrointestinal complications and nutritional status in postoperative patients with esophageal cancer. This scheme will be written according to the Consolidated Standards of Reporting Trials,^[14]^ and the Consolidated Standards of Reporting Trials flow chart is shown in Figure 1.

### 2.2 Ethics and registration

This protocol conforms to the Helsinki Declaration and has been approved by the Clinical Research Ethics Committee of our hospital. This experiment has already been registered (OSF)(registration number: DOI 10.17605/OSF.IO/QHW86). We will fully inform patients and their families about the potential risks of this study. Patients or their families are required to sign a written informed consent form. Patients are free to choose whether to continue the trial at any time during the study.

### 2.3 Patients

#### 2.3.1 Inclusion criteria

(1) All patients underwent radical resection of esophageal cancer and were confirmed by postoperative pathology.
(2) The patient has no cognitive impairment and can communicate normally.
(3) Patients and their families voluntarily participated in the study and signed the informed consent form.

#### 2.3.2 Exclusion criteria

(1) Previous history of gastrointestinal surgery, history of inflammatory enteritis and recurrence of esophageal cancer.
(2) Those who suffer from severe metabolic diseases or who have developed severe cachexia.
(3) Patients with severe liver, kidney, lung function diseases and immune system diseases.
(4) Had a long history of hormone use before operation.
(5) Patients who took probiotics by themselves before operation.

### 2.4 Sample size

The sample size was calculated based on the score of serum albumin six weeks after surgery. According to the results of the pilot study, the score of the probiotics group was 37.94 ± 7.15 and that of the placebo group was 34.53 ± 5.29. Set α = 0.025, one-tailed, and *β* = 0.10. According to the calculation of PASS15.0 software, each group needs 86 participants, and the estimated withdrawal rate is 10%, and 96 participants will be included in each group.

### 2.5 Program implementation

We will screen patients who meet the criteria through pre-hospital recruitment and in-hospital inpatients. 192 patients will be included in this study. Patients will randomly select any number from 001 to 192 from a sealed envelope and are assigned to the probiotic group (group A) and placebo group (group B) according to the parity of the number. Probiotics and placebo are marked “A” and “B” respectively. The probiotics are Live Combined Bifidobacterium, Lactobacillus and Enterococcus Powder (Shanghai Xinyi Pharmaceutical Co. Ltd. National drug license S10970105, 1 g/bag), made up of Bifidobacterium, Lactobacillus acidophilus, Enterococcus faecalis. The number of living bacteria per gram is not less than 1.0 × 107CFU. The placebo is made of starch, and there is no difference in appearance and smell between them.

All the patients will be operated by the same operation team. There is no obvious anastomotic fistula which is confirmed by upper gastrointestinal radiography after the operation. They can start eating. On the advice of the dietitian, the patients in the two groups will be given a liquid diet and gradually transferred to a semi-fluid diet to a normal diet. To ensure that antibiotics given after surgery do not interfere with the results of the study, patients will start taking probiotics or a placebo two weeks after surgery. The dose is 2 bags per time, 3 times a day, and infused with warm water for 8 weeks.

### 2.6 Observation index

(1) Nutritional status index: fasting venous blood will be taken from the two groups at 2 week, 6 weeks after operation and at the end of treatment to detect hemoglobin (Hb), serum albumin, prealbumin and total lymphocyte count, and to measure the height and weight of the two groups at two time points. Calculate body mass index, body mass index normal value > 20.0–25.0 kg/m^2^, 18.5∼20.0 kg/m^2^ is potential malnutrition < 18.5 kg/m^2^ is malnutrition.
(2) Complication index: anorexia, acid regurgitation, nausea, vomiting, diarrhea (≥3 times/d), pulmonary infection, incision infection and anastomotic leakage will be recorded during the study period.

### 2.7 Data collection and management

The patients are followed up by telephone for 30 days after treatment by two assistants, and the data will be collected and recorded. The relevant information and data of this study will be collected, shared and stored in a separate storeroom to protect the confidentiality before, during and after the test. People outside this research group do not have access to relevant data.

### 2.8 Statistical analysis

The collected data are statistically analyzed by SPSS 18.0 software. The counting data use the chi-square test; measurement data use the mean ± standard deviation ($\bar{x}\pm s$), the normal distribution uses the independent sample t-test, and the skewed distribution uses the Mann-Whitney *U* test. When *P* *<0.05*, the difference is considered to be statistically significant.

## 3 Discussion

After operation, the gastrointestinal tract of patients with esophageal cancer is mostly in a state of hypodynamic, and up to 50% of patients will have disturbance of gastric emptying, gastric dumping syndrome, dysphagia and other phenomena, which can lead to enteral nutritional intolerance, adverse reactions such as reflux, vomiting, abdominal distension, constipation, diarrhea, and even serious complications such as aspiration pneumonia and severe malnutrition.^[15]^ Due to perioperative fasting, gastrointestinal mucosal cells lack of food stimulation for a long time, ischemia and hypoxia lead to the decrease of mucosal cell proliferation and repair ability, the weakening of barrier function, and the weakening of immune defense.^[16]^ Intestinal bacterial translocation^[17]^ and increased endotoxin secretion^[18]^ are more likely to occur, and the probability of enterogenous infection increases. The high metabolism caused by surgical destruction of the mucosal barrier and stress is prone to acid-base imbalance, electrolyte disturbance, early postoperative gastrointestinal intolerance and so on to further aggravate the imbalance of gastrointestinal flora.

Some studies have pointed out that probiotics can maintain the balance of intestinal flora, reduce ectopic flora and endotoxin into the blood,^[19]^ and regulate intestinal motility in both ways, which can alleviate the severity of diarrhea in patients with irritable bowel syndrome with hyperkinetic bowel syndrome. It can also reduce the degree of abdominal distension caused by gastrointestinal motility deficiency, increase the frequency of intestinal peristalsis, maintain intestinal peristaltic continuity,^[20]^ and effectively improve the nutritional status of postoperative patients with digestive system tumors. It is beneficial to the recovery after operation.^[21]^ Supplementation of probiotics can reduce the incidence of gastrointestinal complications and improve the nutritional status of patients after surgery by regulating gastrointestinal flora, improving microecological balance and restoring the physiological function of gastrointestinal tract in tumor patients.^[22]^ At present, there are few clinical studies on the application of probiotics for postoperative patients with esophageal cancer, and it is still controversial whether probiotics is beneficial to postoperative patients with esophageal cancer. Therefore, we intend to use this randomized controlled study to explore the effects of probiotics on gastrointestinal complications and nutritional status in postoperative patients with esophageal cancer.

The regionalization of the study population and short follow-up time are the shortcomings of this study program. Therefore, more multi-center and long-term follow-up studies are needed to determine the effects of probiotics on postoperative patients with esophageal cancer.

## Author contributions

**Conceptualization**: Chao Liu and Jing Yang.

**Data curation**: Chao Liu and Jing Yang.

**Formal analysis**: Weiwei Dong.

**Funding acquisition**: Jinyan Yuan.

**Software**: Jing Yang and Weiwei Dong.

**Supervision**: Jinyan Yuan, Weiwei Dong.

**Writing – original draft:** Chao Liu and Jing Yang.

**Writing – review & editing**: Chao Liu and Jinyan Yuan.
